# Human Milk Composition Is Associated with Maternal Body Mass Index in a Cross-Sectional, Untargeted Metabolomics Analysis of Human Milk from Guatemalan Mothers

**DOI:** 10.1016/j.cdnut.2024.102144

**Published:** 2024-04-10

**Authors:** Kasthuri Sivalogan, Donghai Liang, Carolyn Accardi, Anaite Diaz-Artiga, Xin Hu, Erick Mollinedo, Usha Ramakrishnan, Sami Nadeem Teeny, ViLinh Tran, Thomas F Clasen, Lisa M Thompson, Sheela S Sinharoy

**Affiliations:** 1 Nutrition and Health Sciences, Laney Graduate School, Emory University, Atlanta, Georgia, USA; 2 Hubert Department of Global Health, Rollins School of Public Health, Emory University, Atlanta, GA, United States; 3 Gangarosa Department of Environmental Health, Rollins School of Public Health, Emory University, Atlanta, GA, United States; 4 Clinical Biomarkers Laboratory, Department of Medicine, Emory University, Atlanta, GA, United States; 5 Center for Health Studies, Universidad del Valle de Guatemala, Guatemala City, Guatemala; 6 Department of Environmental Health, College of Public Health, University of Georgia, Athens, GA, United States; 7 Nell Hodgson Woodruff School of Nursing, Emory University, Atlanta, GA, United States

**Keywords:** untargeted high-resolution metabolomics, human milk, body mass index, dietary diversity

## Abstract

**Background:**

Maternal overweight and obesity has been associated with poor lactation performance including delayed lactogenesis and reduced duration. However, the effect on human milk composition is less well understood.

**Objectives:**

We evaluated the relationship of maternal BMI on the human milk metabolome among Guatemalan mothers.

**Methods:**

We used data from 75 Guatemalan mothers who participated in the Household Air Pollution Intervention Network trial. Maternal BMI was measured between 9 and <20 weeks of gestation. Milk samples were collected at a single time point using aseptic collection from one breast at 6 mo postpartum and analyzed using high-resolution mass spectrometry. A cross-sectional untargeted high-resolution metabolomics analysis was performed by coupling hydrophilic interaction liquid chromatography (HILIC) and reverse phase C18 chromatography with mass spectrometry. Metabolic features associated with maternal BMI were determined by a metabolome-wide association study (MWAS), adjusting for baseline maternal age, education, and dietary diversity, and perturbations in metabolic pathways were identified by pathway enrichment analysis.

**Results:**

The mean age of participants at baseline was 23.62 ± 3.81 y, and mean BMI was 24.27 ± 4.22 kg/m^2^. Of the total metabolic features detected by HILIC column (19,199 features) and by C18 column (11,594 features), BMI was associated with 1026 HILIC and 500 C18 features. Enriched pathways represented amino acid metabolism, galactose metabolism, and xenobiotic metabolic metabolism. However, no significant features were identified after adjusting for multiple comparisons using the Benjamini–Hochberg false discovery rate procedure (FDR_BH_ < 0.2).

**Conclusions:**

Findings from this untargeted MWAS indicate that maternal BMI is associated with metabolic perturbations of galactose metabolism, xenobiotic metabolism, and xenobiotic metabolism by cytochrome p450 and biosynthesis of amino acid pathways. Significant metabolic pathway alterations detected in human milk were associated with energy metabolism-related pathways including carbohydrate and amino acid metabolism.

This trial was registered at clinicaltrials.gov as NCT02944682.

## Introduction

Maternal obesity has been linked to the increased risk for adverse maternal and fetal outcomes, including gestational diabetes mellitus (GDM), hypertensive disorders of pregnancy, cesarean delivery, miscarriage, stillbirth, preterm birth, and fetal macrosomia [1,2]. Maternal obesity is also one of the strongest predictors of childhood obesity, increasing risk by more than 2-fold, and accounts for 12%–15% of the population attributable risk of childhood obesity [[3], [4], [5]]. Several studies have indicated that maternal overweight and obesity is associated with changes in infant feeding practices, including delayed lactogenesis and reduced duration of feeding [6,7]. Human milk has a dynamic composition of nutrients and bioactive compounds, including enzymes, hormones, cytokines, and more, and varies in composition within a feeding, diurnally, over the entire course of lactation and between mothers and populations [8]. Animal models demonstrate that maternal obesity and high-fat diets negatively impact milk fat production, by blunting the diurnal fluctuation in metabolism, suppressing induction of carbohydrate utilization and altering trafficking of dietary and de novo–derived calories to the mammary glands, impacting energy balance and fuel utilization [9].

However, gaps remain in our understanding of metabolic consequences between maternal overweight and obesity and milk composition. Epidemiological studies have produced contradictory results because it is challenging to disentangle the effects of certain exposures on human milk composition from other prenatal and maternal exposures, as well as other infant feeding practices [10,11]. It has been hypothesized that milk from mothers with obesity may contain different amounts of obesogenic components, compared with protective components [12]. A meta-analysis identified a 0.56 g/L increase in milk fat for every 1 kg/m^2^ increase in maternal prepregnancy BMI, translating to an estimated 16.5% increase in milk fat for a mother with a BMI of 30 compared with a mother with a BMI ≤ 18.5 [13].

High-resolution metabolomics (HRM) is an analytic technique to identify a broad spectrum of endogenous metabolites, food and microbiome metabolites, and environmental chemical exposures [14,15]. Untargeted HRM identifies thousands of metabolites across different metabolic pathways, can provide relative metabolic quantification, and enables investigations of association between external exposure and endogenous processes or adaptations, as well as hypothesis generation for future studies [16]. For example, an untargeted analysis of human milk samples from 94 females at 6 mo postpartum identified enriched monosaccharides and sugar alcohols in milk from mothers with obesity, suggesting that maternal BMI may influence the maturation of transitional milk to mature milk [17]. Traditional composition studies have linked maternal BMI to differences in lipid composition (a higher proportion of SFAs and lower n–3:n–6 PUFA ratio), increased milk content of insulin, leptin, C-reactive protein, IL-6, and TNF-α, lower adiponectin and differences in oligosaccharide composition [1,18,19]. Investigations into the role of maternal BMI or obesity on associated changes in human milk composition, largely via targeted metabolome-associated changes, have returned mixed results and indicate the need for broader untargeted studies [20].

We conducted an untargeted HRM analysis of human milk samples from 75 mothers in Jalapa, Guatemala to expand our understanding of the metabolic consequences of maternal overweight and obesity on human milk composition. This cross-sectional analysis was nested in a randomized control trial (RCT) that investigated the effect of a cleaner cookstove and fuel intervention on health outcomes in pregnant females and their infants. We hypothesized that mothers with overweight and/or obesity would have metabolic perturbations associated with inflammation and oxidative stress compared with normal weight mothers.

## Methods

### Study population

Data were collected from participants who were enrolled in the HAPIN (Household Air Pollution Intervention Network) trial in Guatemala. Detailed information about the trial design and methods has been previously described [21]. HAPIN was a multicenter, parallel-group, individually RCT to determine the effect of a liquefied petroleum gas (LPG) cookstove and free fuel intervention on infant health outcomes including low birth weight, severe pneumonia, and stunting in the first year of life [21]. HAPIN was implemented between May 2018 and September 2021 in ten resource-poor, rural settings of Jalapa, Guatemala; Tamil Nadu, India; Puno, Peru; and Kayonza, Rwanda [21]. The current study utilized data from HAPIN participants residing in rural communities in Guatemala’s Jalapa municipality.

The study has been registered with ClinicalTrials.gov (NCT02944682). The study protocol was reviewed and approved by institutional review boards or ethics committees at Emory University (00089799), Universidad del Valle de Guatemala (146-08-2016/11-2016), and the Guatemalan Ministry of Health National Ethics Committee (11-2016).

### HAPIN eligibility

Pregnant females 18 to <35 y of age, with a confirmed single viable pregnancy between 9 and <20 weeks of gestation, were eligible to participate in the HAPIN trial if they lived in the study area, predominately used biomass stoves (for example, wood and animal dung or charcoal) and provided written informed consent [21]. Pregnant females were excluded if they smoked cigarettes or other tobacco products, planned to move outside of the study area, or used or were likely to predominately use an LPG stove in the near future [21].

### HAPIN randomization and trial group assignment

After eligibility and consent, enrolled females were randomly assigned in a 1:1 ratio to the intervention (LPG stove with continuous fuel supply for ∼18 mo, education and behavior-based messaging to promote safe, exclusive use of the LPG cookstove) or control arms throughout the ∼15-mo recruitment period [21]. Females in the control arm were expected to follow routine cooking practices but received compensation to minimize loss to follow-up and offset the economic advantage accorded to intervention households. Blinding at the participant and field staff level was not possible [21].

### HAPIN data collection

Detailed information about data collection for HAPIN participants has been previously described [21]. In brief, pregnant females were followed through pregnancy and the first year of their infant’s life [21]. This analysis used data collected at baseline, including maternal anthropometry, demographic characteristics, and data collected at 6 mo postpartum, including dietary diversity and milk samples.

### Biosample collection and processing

Within HAPIN, we conducted a pilot substudy to differentiate the infant microbiome and maternal metabolome in rural Guatemala infants and their mothers relative to household air pollution exposure. HAPIN-Guatemala mothers who gave birth between February and August 2019 were recruited from the hospital postpartum ward following birth for the collection of nasal and oropharyngeal samples at birth and milk samples at 6 mo after birth using a convenience sampling approach. Mothers were excluded from participation if they delivered by cesarean section and if the mother reported that she did not intend to nurse her infant at the birth visit. Mothers were further excluded from human milk sampling at the 6-mo visit if they had mastitis, had used antibiotics in the past 48 h, or were no longer nursing. Mastitis was determined by self-report and visual observation by a female surveyor at the time of collection.

Milk samples were collected by a trained Guatemalan female surveyor in the privacy of the participant’s home using a standard protocol for aseptic collection at the same time in the morning (±2 h) for all participants. Nurses showed mothers how to clean the nipple and surrounding area before initiating pumping via breast pump or manual expression. Mothers were asked to fully empty one breast to ensure full expression of foremilk and hindmilk. Milk was expressed into a sterile collection bottle and the aliquot of expressed milk was poured back into 2 sterile tubes. Samples were stored in a −20°C frost-free freezer and then transported to the Universidad del Valle de Guatemala laboratory where they were stored in a −80°C freezer. Samples were shipped on dry ice to Emory’s School of Nursing Research Lab and then transferred to the Clinical Biomarker Laboratory at Emory University for metabolomics profiling. Prediabetes or diabetes status was assessed among females via self-report in a survey questionnaire.

#### Maternal anthropometry

Baseline height and weight were measured at the first baseline visit when mothers were in their first or second trimester of pregnancy. Maternal height was measured using a Seca 213 stadiometer (seca GmbH) in centimeters to the nearest 0.1 cm. Maternal weight was measured using a Seca 876 scale (seca GmbH) and measured to the nearest 0.1 kg. Two measurements for height and weight were taken. If the first 2 measurements for height and weight had a difference of >1 cm (height) or 0.5 kg (weight), then a third measurement was taken, and the 2 closest measurements were used to calculate average height and weight. Maternal BMI was operationalized as a continuous variable because baseline maternal BMI measurements are not reflective of prepregnancy weight and BMI. Traditional BMI categories (underweight, normal, overweight, and obese) were not calculated given that BMI was based on weight measurements at the baseline assessment when participants were late in their first, or early in their second, trimester of pregnancy.

#### Covariates

Maternal age and education were assessed at baseline, whereas maternal dietary diversity was measured at 6 mo postpartum. Maternal education was categorized as follows: *1*) no formal education or some primary school, *2*) primary school or some secondary school, or *3*) secondary, vocational, or some university. Maternal dietary diversity was assessed using the tool adapted from the FAO of the United States to calculate Minimum Diet Diversity for Women [22]. Females were asked to recall the consumption of a prespecified list of food groups in the past 24 h at baseline and at 6 mo postpartum, which were then categorized into 10 categories including grains, white roots, tubers, and plantains; pulses; nuts and seeds; dairy; meat, poultry, and fish; eggs; dark green leafy vegetables; other vitamin A-rich fruits and vegetables; other vegetables; and other fruits. Individual consumption was summed into a score ranging from 0 to 10 based on yes/no responses to the prespecified food groups and each woman was classified as achieving dietary diversity using a cutoff value of ≥5 [23].

### High resolution metabolomics

We followed an established untargeted metabolome-wide association study (MWAS) workflow, which included 4 major steps: *1*) metabolomics profiling, *2*) statistical analysis, *3*) pathway enrichment analysis, and *4*) metabolite identification [15,[24], [25], [26], [27]].

#### Metabolomics profiling

Human milk samples were analyzed in triplicates across 4 batches using liquid chromatography coupled with high-resolution tandem mass spectroscopy (LC-MS) following an established protocol [28,29]. Two chromatography columns were used to enhance the coverage of metabolic features detection—the hydrophilic interaction liquid chromatography (HILIC) with positive electrospray ionization (ESI) and reverse phase (C18) chromatography with negative ESI. A pooled milk sample that was referenced against the National Institute of Standards and Technology standard reference material was added to each batch. Quality control reference samples were included at the beginning and end of each analytic batch for normalization, control for background noise, batch evaluation, and post hoc quantification.

Data were extracted using an adaptive apLCMS R package (Tianwei Yu, version 6.6.9) which performs peak detection, noise removal, peak quantification, peak alignment, and recovery of weak signals [30]. Another R package, xMSanalyzer (version 2.0.6.1) was used to optimize peak detection by merging results from different parameter settings, quality evaluation of samples and features, mass-to-charge ratio (*m/z*) calibration using internal standards and confirmed metabolites, and *m/z* calibration using internal standards and confirmed metabolites and batch-effect evaluation and correction [31]. Detected signals are known as metabolic features and are uniquely defined by *m/z*, retention time, and ion intensity.

#### Statistical analysis

Descriptive baseline analyses of the 75 study participants who provided milk samples were conducted using SAS/STAT (version 9.4). We investigated the association of continuous maternal BMI with milk metabolic feature intensities using multivariate linear regression. In the following model:

Model 1: *Y*_*ij*_ *= β*_0_ *+ β*_1_ (BMI) *+ β*_2_ (Maternal age) *+ β*_3_ (Maternal education) *+ β*_3_ (Maternal dietary diversity) *+ ε*_*ij*_

*Y*_*ij*_ refers to the log_2_ intensity of metabolic feature *j* for participant *I*, *β*_*0*_ is the intercept, which refers to the predicted value of the log_2_ intensity of metabolic feature when setting continuous variables to zero and categorical variables to their referent value. *ε*_*ij*_ is the residual random normal error and BMI_*i*_ refers to the continuous maternal BMI for participant *i*.

**FIGURE 1:**
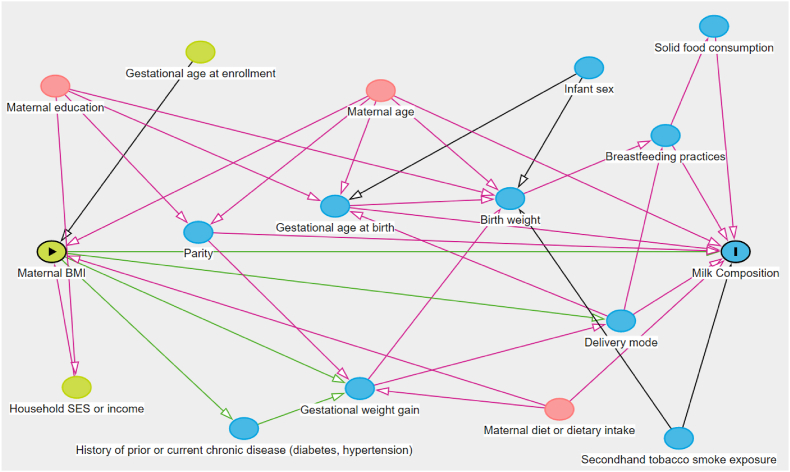
Directed acyclic graph to identify confounders associated with maternal BMI and milk composition. Minimally sufficient adjustment sets for estimating the effect of maternal BMI (exposure) on milk composition (outcome) are highlighted in pink. Green arrows represent causal paths and pink arrows represent biasing paths. Yellow circles represent ancestors of exposure (maternal BMI), whereas blue circles represent ancestors of outcome (milk composition). Pink circles are ancestors of exposure and outcome.

Covariates and potential confounding factors were identified a priori, from a review of existing literature and use of a directed acyclic graph (DAG) (Figure 1), which include maternal age, maternal education, and maternal dietary diversity in the adjusted model.

Metabolomics analyses were performed using R (version 4.2.1), and statistical significance was defined at *α* < 0.05. The untargeted MWAS examined the association between maternal BMI and global metabolic signals and pathways without a priori knowledge of chemical identities. Separate MWAS models were built for each metabolic feature from each dataset (HILIC/+ESI and C18/-ESI) [32]. The FDR was controlled using the Benjamini–Hochberg procedure to correct for multiple comparisons [33]. However, a limited number of significant features were detected when using the FDR, due to the small sample size used in this pilot study, and thus, a less conservative corrected *P* value threshold (FDR < 0.2) was used to decrease the possibility of false negatives. FDR < 0.2 was selected as the threshold to be able to generate a novel hypothesis because 0.2 is consistent with what has been commonly used in the field of environmental metabolomics [34].

#### Pathway enrichment

Pathway analyses predict biological functions and molecular mechanisms associated with significant metabolic features. We used both Mummichog (version 1.0.9) and Metapone (version 3.18) for the pathway testing to infer and categorize functional biological activity directly from mass spectrometry output. Mummichog (version 1.0.9) is used to infer functional activity and metabolic pathways and networks without prior chemical identification [35]. The algorithm uses *m/z* values to match possible metabolites to metabolic features and construct pathways based on tentative identification. Significant features (raw *P* < 0.05 by limma test) were further studied by pathway enrichment analyses using Mummichog (version 1.0.9). This approach protects against type 2 statistical error by including all features at *P* < 0.05 and protects against type 1 statistical error by permutation testing in pathway enrichment analysis. Metapone (version 3.18) is a newer method that can jointly analyze positive- and negative-ion mode data to generate a more integrated and comprehensive view of the metabolic perturbations and avoid double counting [36].

#### Metabolite identification

Significant features were matched to the METLIN Metabolite and Chemical Entity Database, ChemSpider, Human Metabolome Database, and Kyoto Encyclopedia of Genes and Genomes using a mass error threshold of 5 ppm [31]. Chemical identities were confirmed by manual visual inspection of extracted ion chromatographs for all significant metabolites. To distinguish true peaks from noise signals and minimize false positives, peaks must have exhibited clear Gaussian peak shapes and a signal-to-noise ratio greater than 3:1. True metabolic features were then confirmed with level 1 confidence using Metabolomics Standards Initiative criteria of features whose *m/z* (±10 ppm difference) and retention time (±10 s) matched the authentic compounds [30]. Level 1 evidence indicates that the proposed metabolites have been confirmed via appropriate measurement of an authentic reference standard with the same experimental conditions as the milk samples. Metabolic features not assigned with level 1 confidence were annotated by xMSannotator (version 2.0.6.1) [32].

## Results

### Sample population

Nurses approached all females who delivered at the hospital between February and August 2019 for eligibility in the pilot substudy. Seventy-six mothers were recruited and consented to participate in the pilot from the intervention group and 72 mothers consented to participate in the pilot from the control group.

**FIGURE 2:**
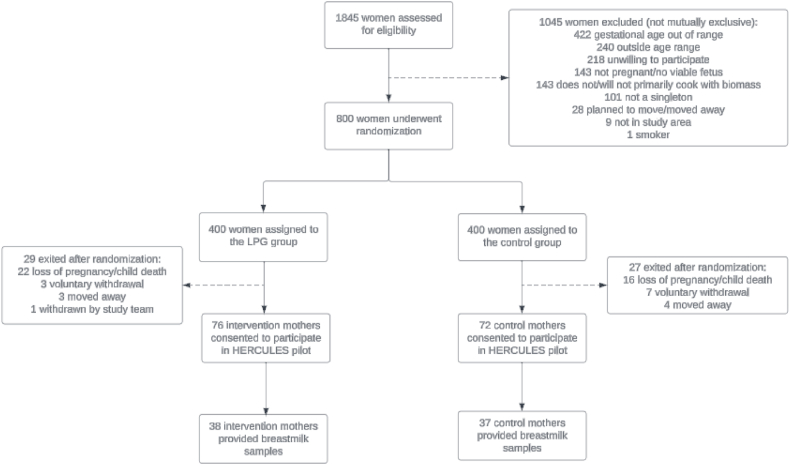
Participant flow chart for identifying *n* = 75 sample size for breast milk sample collection. LPG, liquefied petroleum gas.

However, based on budgetary constraints, a sample size of 75 was predetermined for human milk sample collection and analysis. A total of 38 mothers from the HAPIN intervention and 37 mothers from the HAPIN control groups provided milk samples at the 6 mo postpartum household visit (Figure 2).

### Population characteristics

Characteristics of the study population (Table 1) were similar to those of the larger HAPIN-Guatemala cohort (Supplemental Table 1) [37]. The mean age of participants at baseline was 23.62 ± 3.81 y, whereas the mean gestational age of participants at enrollment was 14.64 wk ± 2.88 wk. The mean BMI was 24.27 ± 4.22 kg/m^2^. However, there was a wide range of BMI measurements in this sample with the median at 23.32 kg/m^2^ (IQR: 21.71–26.08 kg/m^2^). In addition, no females had prediabetes or diabetes at baseline.

**TABLE 1:** Sociodemographic characteristics of study population

Study participants (*n* = 75)	
Maternal age (y), *n* (%)	
<20	15 (20.00)
20–24	33 (44.00)
25–29	20 (26.67)
30–35	7 (9.33)
Maternal height (cm), mean (SD)	147.68 (±7.47)
Maternal BMI (kg/m^2^) mean (SD)	24.27 (±4.22)
Maternal highest level of education completed, *N* (%)	
No formal education/some primary school	29. (38.67)
Primary school/some secondary school	33 (44.00)
Secondary/vocational/some University	13 (17.33)
Maternal minimum dietary diversity (score), *N* (%)	
Low (<4)	73 (97.33)
High (≥5)	2 (2.67)
Household food insecurity (score), *N* (%)	
Food secure	39 (52.70)
Mild (1, 2, and 3)	25 (33.78)
Moderate (4, 5, and 6)/severe (7 and 8)	10 (33.78)
No. of people sleeping in house, mean (SD)	5.29 (±2.29)
Gestational age at enrollment (wk), mean (SD)	14.64 (±2.88)
Preterm, *N* (%)	
Yes	3 (4.00)
Nulliparous, *N* (%)	
Yes	18 (24.0)
Delivery mode, *N* (%)	
Vaginal	59 (78.67)
Cesarean	16 (21.33)
Reported exclusive breast feeding at 6 mo, *N* (%)	70 (93.33)

Diet diversity was low at baseline (mean: 2.65, 95% CI: 2.45, 2.86) and remained low at 6 mo postpartum (mean: 2.81; 95% CI: 2.56, 3.06). Diet diversity was almost uniformly lacking in diversity (with 97% consuming fewer than 5 food groups per day). The majority reported consuming grains and legumes daily, with over half reporting weekly or monthly consumption of chicken or eggs and less than half reporting weekly or monthly consumption of dark greens and fruit.

### Metabolome changes associated with BMI

**FIGURE 3:**
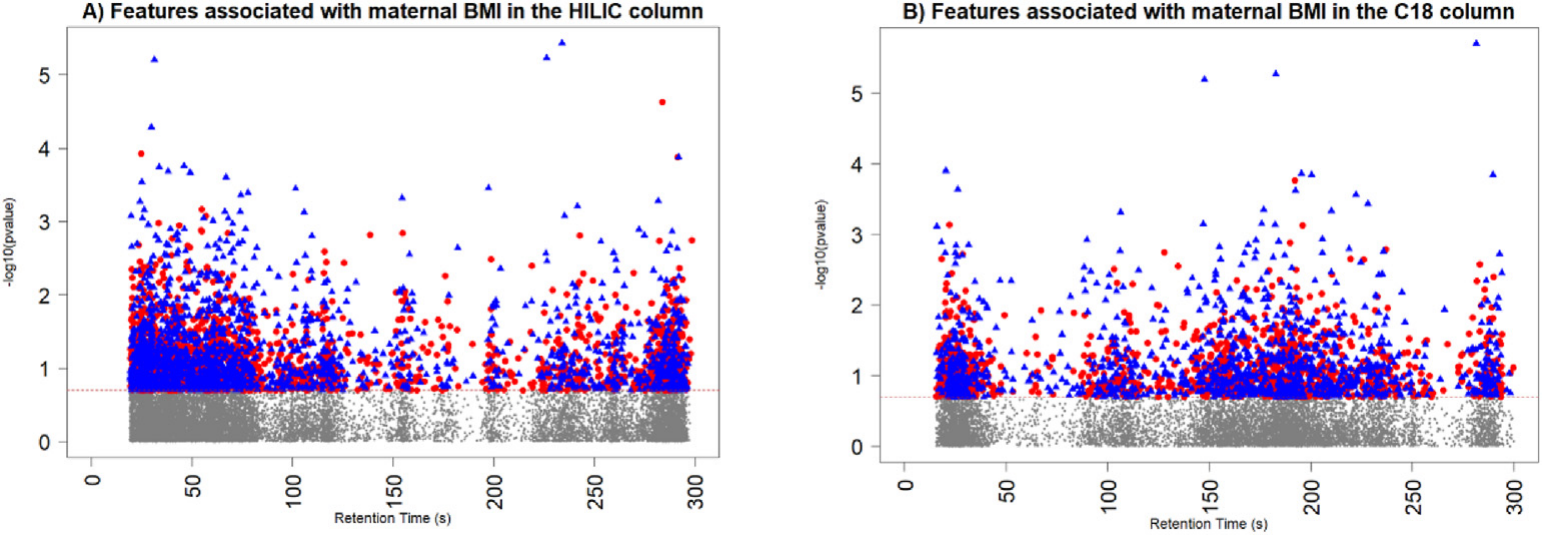
Manhattan plot of the association between changes in metabolic features associated with continuous maternal BMI. The x-axis presents the retention time of the metabolic features, and the y-axis presents the negative natural log of the *P* value in exposure to metabolites association. Red dots represent metabolic features that are positively associated with maternal BMI and blue triangles represent metabolic features negatively associated with maternal BMI. The dashed red line is the threshold-log_10_ (*P* < 0.05). HILIC, hydrophilic interaction liquid chromatography.

After processing and data quality checks, 19,199 metabolic features from the HILIC/+ESI column and 11,594 metabolic features from the C18/−ESI column were included in the final analyses, as can be seen in Figure 3.

Two MWAS models were analyzed using data from each of the chromatography columns. After adjusting for covariates, maternal age, maternal education, and maternal dietary diversity, 1000 *m/z* features were significantly associated with continuous maternal BMI in the HILIC ESI and 590 *m/z* features in the C18 ESI (*P* < 0.05), as can be seen in Figure 3. After adjusting for multiple comparison testing, no significant *m/z* features were identified for either column (FDR_BH_ < 0.2). Below, we present the significant metabolic pathways and metabolites identified from the covariate-adjust raw model.

### Pathways enrichment analysis

Metapone identified 8 metabolic pathways that were significantly associated with maternal BMI including galactose metabolism, biosynthesis of amino acids, xenobiotics metabolism and metabolism of xenobiotics by cytochrome p450, fructose and mannose metabolism, C5-branched dibasic acid metabolism, mineral absorption, and serotonergic synapse (Supplemental Figure 1). Significant pathways, number of significant metabolites, and putative identification of significant metabolites associated with maternal BMI are presented in Table 2.

**TABLE 2:** Significant pathways, number of significant metabolites, and putative identification of significant metabolites associated with maternal BMI

Pathway name	*P* value	No. of significant metabolites	No. of mapped metabolites	No. of metabolites	HMDB metabolites	Putatively identified metabolite
Galactose metabolism	<0.001	3	14	56	HMDB0000143 HMDB0000247 HMDB0000169 HMDB0000211 HMDB0006790 HMDB0003213 HMDB0000660 HMDB0000107 HMDB0000122/HMDB0000660 HMDB0003345 HMDB0060468 HMDB0000516 HMDB0001542	D-Galactose Sorbitol D-Mannose Myo-Inositol Galactosylglycerol Raffinose D-Fructose Galactitol D-Glucose Alpha-D-Glucose D-Gal alpha 1->6D-Gal alpha 1->6D-Glucose D-Glucose N-Acetyllactosamine
Metabolism of xenobiotics by cytochrome p450	<0.001	6	33	96	HMDB0062469 HMDB0062397 HMDB0060330/HMDB0060329 HMDB0060327/HMDB0060326 HMDB0060448 HMDB0060446 HMDB0032059 HMDB0011603 HMDB0062402 HMDB0062407	Benzo[a]pyrene 4-Bromophenol 1-Nitro-7-hydroxy-8-glutathionyl-7,8-dihydronaphthalene 1-Nitro-5-hydroxy-6-glutathionyl-5,6-dihydronaphthalene Bromobenzene-3,4-oxide Bromobenzene-2,3-oxide 2-Bromophenol 4-(Methylnitrosamino)-1-(3-pyridyl)-1-butanone 4-Hydroxy-1-(3-pyridinyl)-1-butanone 5-(3-Pyridyl)-2-hydroxytetrahydrofuran
Xenobiotics metabolism	0.005	5	23	76	HMDB0062469 HMDB0060448 HMDB0000806 HMDB0060446 HMDB0032059 HMDB0062397 HMDB0060330/ HMDB0060329 HMDB0060326/HMDB0060327	Benzo[a]pyrene Bromobenzene-3,4-oxide Myristic acid Bromobenzene-2,3-oxide 2-Bromophenol 4-Bromophenol 1-Nitro-7-hydroxy-8-glutathionyl-7,8-dihydronaphthalene 1-Nitro-5-glutathionyl-6-hydroxy-5,6-dihydronaphthalene
Biosynthesis of amino acids	0.005	5	41	92	HMDB0000148 HMDB0000696 HMDB0000159 HMDB0000162 HMDB0000205 HMDB0000904 HMDB0012710 HMDB0003011 HMDB0003665 HMDB0012266 HMDB0000731	Glutamic Methionine Phenylalanine Proline Phenylpyruvic acid Citrulline 3-Dehydroquinic acid O-Acetylserine Phosphoribosyl-ATP N-Succinyl-2-amino-6-ketopimelate Cysteine-S-sulfate
Fructose and mannose metabolism	0.01	1	5	34	HMDB0000169 HMDB0000122 HMDB0000247 HMDB0003345 HMDB0000765 HMDB0003539 HMDB0001151	D-Mannose D-Glucose Sorbitol Alpha-D-Glucose Mannitol Levan Allose
C5-branched dibasic acid metabolism	0.02	2	8	21	HMDB0002092 HMDB0000148 HMDB0029433 HMDB0000634	Itaconic acid Glutamic acid l-2-Amino-4-methylenepentanedioic acid Citraconic acid
Mineral absorption	0.035	1	8	26	HMDB0000122 HMDB0000696 HMDB0000159 HMDB0000162	D-Glucose Methionine Phenylalanine Proline
Serotonergic synapse	0.035	2	12	38	HMDB0000472 HMDB0002995 HMDB0002343 HMDB0002311 HMDB0002314 HMDB0002265	5-Hydroxy-L-tryptophan 12-Keto-tetrahydro-leukotriene B4 5,6-DHET 8,9-DiHETrE 11,12-DiHETrE 14,15-DiHETrE

HMDB, Human Metabolome Database.

Pathway enrichment analyses were analyzed separately in Mummichog (version 1.0.9) for significant metabolic features identified in the HILIC/+ESI and C18/−ESI columns. Sixteen metabolic pathways, from the HILIC/+ESI column, and 12 metabolic pathways, from the C18/−ESI column, were significantly associated with maternal BMI. The Mummichog output for all pathways associated with continuous maternal BMI can be found in Supplemental Table 2. There was no overlap in pathways identified between features detected in the HILIC and C18 modes. Pathways significantly associated with maternal BMI include vitamin groups [thiamin (vitamin B-1)], pyridoxine (vitamin B-6), biotin (vitamin H/B-7), ascorbate (vitamin C), fatty acid metabolism (fatty acid metabolism, fatty acid activation, de novo fatty acid biosynthesis), lipid metabolism (linoleate metabolism, glycosphingolipid and glycosphingolipid-globoseries metabolism), carbohydrate metabolism (hexose metabolism, galactose metabolism, starch and sucrose metabolism), nucleotide metabolism (aminosugar metabolism and purine metabolism), and amino acid metabolism (tyrosine metabolism, purine metabolism, and lysine metabolism).

### Metabolite annotation and identity confirmation

Twenty-eight metabolites associated with BMI were confirmed and identified with level 1 evidence (Table 3). Specifically, 23 metabolites were confirmed identified from the HILIC/+ESI column and 5 metabolites were confirmed identified from the C18/−ESI column. Confirmed metabolites from both columns were related to C5-branched dibasic acid metabolism and were negatively associated with maternal BMI. Metabolites from the HILIC/+ESI column were related to caffeine metabolism and tryptophan metabolism and were positively associated with maternal BMI. In addition, metabolites from both datasets were related to amino acid metabolism including branched-chain amino acid (BCAA) metabolism (valine, leucine, and isoleucine), tyrosine metabolism, cysteine and methionine, phenylalanine, and arginine metabolism. Corresponding parameter estimates from the MWAS models are also presented in Table 3, indicating if the metabolites are positively or negatively associated with maternal BMI.

**TABLE 3:** Chemical identity of level 1 confirmed metabolites significantly associated with continuous maternal BMI (*P* < 0.05)

*m/z*	RT(s)	Identified metabolite	Kegg or HMDB ID	Adduct form	Association with maternal BMI	Pathway or metabolism	ESI
131.03	81.4	2-methylmaleate	C02226	M+H	−0.02881	Valine, leucine, and isoleucine biosynthesis	ESI+
131.03	81.4	Itaconate	C00490	M+H	−0.02881	C5-Branched dibasic acid metabolism	ESI+
138.09	31.1	Tyramine	C00483	M+H	−0.00501	Tyrosine metabolism	ESI+
138.09	31.7	Phenylethanolamine	C02735	M+H	−0.00501	Catecholamine biosynthesis	ESI+
138.09	32	Tyramine/phenylethanolamine	Unknown	M+H	−0.00501	Catecholamine metabolism	ESI+
148.06	77.4	N-methyl-aspartic acid	C12269	M+H	−0.02559	Neurotransmitter and neuroendocrine regulator	ESI+
148.06	42	N-acetylserine	C12269	M+H	−0.02559	Sulfur metabolism	ESI+
150.06	53	Methionine	C00073 C01733	M+H	−0.01017	Cysteine and methionine metabolism	ESI+
165.06	30	4-coumarate	C00811	M+H	−0.01313	Ubiquinone and other terpenoid-quinone biosynthesis	ESI+
165.06	59.2	Phenylpyruvate	C00166	M+H	−0.01313	Phenylalanine metabolism	ESI+
175.09	30.6	Indole-3-acetamide	C02693	M+H	−0.01609	Tryptophan metabolism	ESI+
176.10	91.2	Citrulline	C00327	M+H	−0.01609	Arginine biosynthesis	ESI+
180.09	76.8	Mannosamine	C03570	M+H	−0.01066	Unknown	ESI+
181.08	35	Theophylline	C07130	M+H	0.016358	Caffeine metabolism	ESI+
181.08	36	Paraxanthine	C13747	M+H	0.007511	Caffeine metabolism	ESI+
181.08	36	Theobromine	C07480	M+H	0.007511	Caffeine metabolism	ESI+
181.08	36	Dimethylxanthine	C07480	M+H	0.007511	Caffeine metabolism	ESI+
221.09	58.5	5-hydroxy-tryptophan	C00643	M+H	0.002007	Tryptophan metabolism	ESI+
310.11	76.5	N-acetylneuraminate	C00270	M+H	0.018689	Amino sugar and nucleotide sugar metabolism	ESI+
468.31	42	LPC (14:0)	HMDB0010379	M+H	−0.02186	Phosphatidylcholine turnover	ESI+
508.34	42	LPC (20:1)	HMDB0011512	M+H	0.001738	Phosphatidylcholine turnover	ESI+
538.52	26	Cer(D18:1/16:0)	Unknown	M+H	6.48E-04	Lipid signaling pathway	ESI+
563.27	27.9	Protoporphyrin	4971	M+H	−0.0157	Porphyrin metabolism	ESI+
164.07	25.4	Phenylalanine	C00079	M-H	0.00979	Phenylalanine metabolism	ESI−
173.01	19.3	Transaconitate	C02341	M−H	−0.0012	C5-Branched dibasic acid metabolism	ESI−
181.07	22.2	Galactitol	C01697	M−H	0.00397	Galactose metabolism	ESI−
227.21	220.2	Myristic acid	C06424	M−H	−0.01742	Fatty acid biosynthesis	ESI−
277.22	223.2	Gamma-linolenic acid	C06426	M−H	−0.00259	Linoleic acid metabolism	ESI−

Abbreviations: ESI, electrospray ionization; HMDB, Human Metabolome Database; LPC, lysophosphatidylcholines; RT, retention time.

### Sensitivity analysis

Sensitivity analyses were conducted to reduce false discoveries and to confirm the consistency of the results in our analyses. Different *P* values, including both raw (*P* < 0.05; *P* < 0.005) and FDR-corrected (FDR < 0.2), were used as different cutoff points for the pathway enrichment analyses [35]. All pathways from both the HILIC and C18 columns that were identified at *P* value of <0.05 were also identified at a more stringent *P* value of <0.005, indicating consistency in our results.

## Discussion

In this exploratory study, we conducted HRM to characterize human milk composition and explore changes in the milk metabolome associated with maternal BMI, from among a subpopulation of Guatemalan mothers participating in the HAPIN trial. This untargeted HRM analysis identified metabolites and metabolic pathways involved in energy metabolism that were significantly associated with maternal BMI, including galactose, biosynthesis of amino acids, and xenobiotic metabolism, including cytochrome p450. This is one of the few untargeted HRM studies in the literature to identify that maternal weight, BMI, or overweight and obesity are significantly associated with the human milk metabolome [1,10,20].

A key finding is that metabolites in the galactose metabolism pathway were associated with maternal BMI. Galactose is a component of lactose and a product of hepatic galactose metabolism [38]. In this analysis, galactitol, a reduction product formed from excess galactose, was positively associated with maternal BMI. Our metabolome-wide study showing alteration in galactose metabolites contributes additional evidence that maternal BMI is associated with changes in galactose metabolism. A secondary analysis of breastfeeding participants with normal weight and obesity identified that metabolites involved in galactose metabolism were enriched (*P* < 0.001) over the first 6 mo among females with obesity and remained significant following FDR correction (*P* = 0.01) [17]. In addition, BMI classification was associated with metabolites involved in galactose metabolism (*P* = 0.001) [17].

Metabolites related to the pathways for biosynthesis of amino acids, including methionine, phenylpyruvate, and citrulline, were negatively associated with maternal BMI. Each of these metabolites is involved in the biosynthesis of arginine, cysteine, methionine, and phenylalanine, meaning that these biosynthetic pathways may be altered or dysregulated among mothers with higher BMIs. A study to differentiate the human milk microbiota in colostrum of mothers with GDM or obesity similarly identified that amino acids biosynthesis pathways were overrepresented in mothers with GDM [39]. In addition, a 2-wk reduced-fat and reduced-sugar dietary intervention during lactation altered amino acid biosynthesis pathways (*P* = 0.005) in the stool microbiome among exclusively nursed infants [40]. Additional maternal dietary interventions including high-fat compared with high-carbohydrate and high-glucose compared with high-galactose diets increased bacterial metabolic pathways involved in amino acid biosynthesis in human milk [40]. Results from the literature, and other omics analyses, indicate the need for multiomics studies to integrate metabolomics and microbiome methods to assess the role of maternal BMI on amino acid-related metabolic pathways and long-term health implications for mothers and infants as a result of dysregulated amino acid metabolic pathways [41].

Xenobiotics are chemicals found in but not produced by the organisms or the ecological system being studied. Some naturally occurring endobiotic compounds can also become xenobiotics when present in the environment at excessive concentrations [42]. Cytochrome p450s are a superfamily of protein enzymes involved in the synthesis and metabolism of a range of internal and extracellular components. In this study, xenobiotics metabolism and xenobiotic metabolized by cytochrome p450 were associated with maternal BMI. Caffeine, one of the most widely used xenobiotic compounds, is metabolized by the cytochrome p450 oxidase enzyme system into paraxanthine, theobromine, and theophylline [43]. All L-1 confirmed caffeine metabolites identified in this study, including theophylline, paraxanthine, theobromine, and dimethylxanthine, were positively associated with maternal BMI.

The literature on the role of xenobiotics in human milk is sparse—1 study suggests that variations in diet or dietary patterns may be associated with concentration of xenobiotic metabolites in human milk [1]. Another study examining alterations in urine samples from preterm infants consuming donor human milk compared with their own mother’s milk identified differences in 11 metabolites that were related to metabolism of xenobiotics by cytochrome p450 [44]. Our results add to the literature by providing the first evidence of associations, to our knowledge, among maternal BMI, xenobiotic metabolism, and metabolism of xenobiotics by cytochrome p450.

The pathways described above—galactose metabolism, biosynthesis of amino acids, and xenobiotic metabolism—have been examined in studies of the infant fecal metabolome [45,46]. Fecal metabolites belonging to galactose metabolism were associated with infant age among exclusively nursed infants, whereas biosynthesis of amino acids was enriched in formula-fed infants, compared with nursed infants [45]. Xenobiotics were also identified as the highest milk-associated bacterial compound found in the neonatal fecal metabolome, indicating milk-fecal cross-talk [46]. These results indicate that maternal BMI, maternal diet, and infant diet (namely human milk) may interact in shaping infant health, as reflected in the infant fecal metabolome.

This analysis additionally confirmed the identities of 28 chemical metabolites, as presented in Table 3, which were associated with maternal BMI. As indicated in Table 3, all metabolites, except for caffeine metabolites, were negatively associated with maternal BMI. Our results indicate associations between maternal BMI and milk metabolites predictive of insulin resistance and diabetes risk. We found that tyramine, a monoamine derivative of tyrosine and product of amino acid metabolism, was negatively associated with maternal BMI. Tyrosine is an aromatic amino acid and has been found to be increased in both human milk and plasma of females with obesity and negatively associated with metabolic health [10]. In 1 study among mother–infant dyads, the concentration of tyrosine was 30% higher (*P* = 0.016) in human milk from mothers with obesity, compared with human milk from normal weight mothers [47]. In another study, maternal exposure to nonnutritive sweeteners (NNS) during pregnancy and lactation caused critical changes in amino acid metabolism in NNS pups [48]. Amino acids were the most deregulated class of metabolites with tyramine being highly upregulated [48]. Our results are consistent with the literature as aromatic amino acids have strong predictive value for insulin resistance and type 2 diabetes [49,50]. In addition, tyrosine competes with branched-chain amino acids for l-type amino acid transporter responsible for cell and tissue entry and this could explain the higher tyrosine concentrations found in the plasma of individuals with obesity [51].

In addition to tyrosine, 2-methylmaleate, a precursor to BCAA biosynthesis, is predictive of diabetes risk and associated with diabetes development [52]. In our study, 2-methylmaleate was negatively associated with maternal BMI. A study examining free amino acid concentration in human milk between females with obesity and females with normal weight mothers identified that females with obesity produce human milk that differs from females with normal weight [53]. In addition, free BCAAs were higher in human milk from females with obesity and maternal BMI was positively associated with isoleucine and leucine concentrations over the first 6 mo of infant life [45]. BCAAs are associated with the development of type 2 diabetes mellitus (T2DM) and nonalcoholic fatty liver disease and increased BCAA concentration strongly predict T2DM [53]. Research has suggested targeting BCAA catabolism as a mechanism to treat obesity-associated insulin resistance [54].

Our results also indicate significant associations between maternal BMI and metabolites that may be predictive of future GDM risk [55]. Specifically, we observed associations between maternal BMI and LPC(14:0) and LPC(20:1), which are part of a class of lipids, lysophosphatidylcholines (LPCs), that activate multiple signaling pathways involved in oxidative stress and inflammatory response [55]. LPC(14:0) is associated with obesity risk in the literature [56]. A study to identify BMI-related lipids and the role of lipids linking BMI and GDM identified a relationship between BMI and LPC(14:0) [57]. Furthermore, BMI-associated lipids, including LPC(14:0), were estimated to explain ∼66.4% of the relationship between higher BMI and GDM, suggesting that BMI-related lipids may play a role in GDM pathogenesis [57]. Metabolomics literature on LPCs also indicates associations between LPCs with infant weight gain, childhood overweight, and obesity [58]. Future studies could examine the role of metabolomic LPCs and obesity risk in maternal–infant dyads.

Similarly, we observed a positive association between maternal BMI and a lipid biomarker, Cer(D18:1/16:0), which is one of the most well-studied and validated lipid biomarkers predictive of morbidity and mortality, including GDM [59]. A nested case-control study from a prospective cohort of pregnant females identified that high Cer18:1 was associated with higher GDM risk [60]. Another study demonstrated that Cer18:1 concentration was higher among females who eventually developed GDM, compared with females with normal glucose tolerance [61]. Cer18:1 concentrations were elevated in pregnancy and changes were not independent of BMI [61]. Because increased maternal overweight or maternal obesity is associated with increased GDM risk, it is conceivable that Cer18:1, enriched in females with GDM risk or developed GDM, would also be associated with maternal BMI.

The main strength of this analysis is the use of cutting-edge untargeted HRM to investigate biological mechanisms associated with differences in maternal BMI in a cohort of Guatemalan mothers. This analysis is unique in the use of human milk as a nonintrusive biological tool to examine changes in the milk metabolome from females living in a rural environment. Although smaller than epidemiological studies, the sample size for this analysis is comparable with other hypotheses generating untargeted HRM in the literature [62,63]. In addition, multiple comparison testing, and sensitivity analyses were conducted to reduce risk of false positive discoveries.

There are a few limitations that must be considered in the interpretation of our findings. First, the single time point sampling and cross-sectional analysis neither allow us to consider the effect of the known variations in human milk composition, such as lactation phase and variation within and between feeds nor would we be able to make any causal inference. Second, this analysis was conducted with a small sample size. Although this is common in hypothesis-generating metabolomics studies, the analysis was not well-powered to detect a significant difference after adjusting for multiple comparisons. Third, maternal BMI was only measured in the first and second trimesters of pregnancy for all mothers and may not reflect prepregnancy BMI or postpregnancy weight gain. However, Retnakaran et al. [64] prospectively evaluated the relationship between pregravid weight and first trimester weight. The authors recognize that pregravid and first trimester weight measurements are unlikely to be identical due to modest physiological weight gain in the first trimester [64]. However, there was good concordance between weight measurements suggesting that first trimester weight measurement can be a reasonable surrogate for pregravid weight, with the caveat that differences between these measurements are generally modest but can vary in magnitude [64]. Although all mothers in this study sample were nursing at 6 mo postpartum, we did not collect additional information on infant feeding practices. This study population was conveniently sampled from the larger Guatemalan HAPIN cohort, so the results from this analysis may not be generalizable to the full cohort or to a broader population. There is a risk of selection bias as mothers were sampled in postpartum wards following the birth of the infant, but this study sample is demographically similar to the larger HAPIN-Guatemala cohort, as presented in Supplemental Table 1 [37]. Although a DAG was used to identify and control for significant covariates, potentially important confounders related to maternal BMI (such as patterns of infant feeding) were not collected or included in this analysis. Despite the limitations, this study contributes to the growing literature on human milk composition using untargeted metabolomics.

## Acknowledgments

We would like to thank the members of the advisory committee—Patrick Breysse, Donna Spiegelman, and Joel Kaufman—for their valuable insight and guidance throughout the implementation of the trial. We would also like to thank all research staff, including Maria Renee Lopez and John P. McCracken, and study participants for their dedication to and participation in this important trial. We are grateful to Jiantong Wang for data management and processing support and to Yilin Wang for analytic support.

## Author contributions

The authors’ responsibilities were as follows – KS: conceptualization, formal analysis, visualization, writing—original draft, writing—review and editing; DL: conceptualization, methodology, software, formal analysis, supervision, writing—review and editing; CA: data curation, methodology, resources, writing—review and editing; AD-A: project administration, supervision, writing—review and editing; XH: data curation, methodology; EM: writing—review and editing; UR: supervision, writing—review and editing; SNT: data curation, methodology, writing—review and editing, VT: data curation, methodology, investigation, writing—review and editing; TFC: supervision, writing—review and editing; LMT: conceptualization, funding acquisition, project administration, supervision, writing—review and editing; SS: conceptualization, funding acquisition, supervision, writing—review and editing; and all authors: read and approved the final manuscript.

## Conflict of interest

The authors report no conflicts of interest.

## Funding

The HAPIN trial is funded by the United States National Institutes of Health (cooperative agreement 1UM1HL134590) in collaboration with the Bill & Melinda Gates Foundation (OPP1131279). Pilot funds from the HERCULES program in Environmental Health Sciences at Emory University supported the collection of 75 human milk samples for metabolomics profiling. This work was supported by the National Institute of Environmental Health Sciences of the National Institutes of Health under Award Number P30ES019776. The content is solely the responsibility of the authors and does not necessarily represent the official views of the National Institutes of Health.

A multidisciplinary, independent Data and Safety Monitoring Board (DSMB) appointed by the National Heart, Lung, and Blood Institute (NHLBI) monitors the quality of the data and protects the safety of patients enrolled in the HAPIN trial. NHLBI DSMB: Nancy R. Cook, Stephen Hecht, Catherine Karr (Chair), Joseph Millum, Nalini Sathiakumar, Paul K. Whelton, and Gail Weinmann and Thomas Croxton (Executive Secretaries). Program Coordination: Gail Rodgers, Bill & Melinda Gates Foundation; Claudia L. Thompson, National Institute of Environmental Health Sciences; Mark J. Parascandola, National Cancer Institute; Marion Koso-Thomas, Eunice Kennedy Shriver National Institute of Child Health and Human Development; Joshua P. Rosenthal, Fogarty International Center; Concepcion R. Nierras, NIH Office of Strategic Coordination Common Fund; Katherine Kavounis, Dong- Yun Kim, Antonello Punturieri, and Barry S. Schmetter, NHLBI. The findings and conclusions in this report are those of the authors and do not necessarily represent the official position of the US National Institutes of Health or Department of Health and Human Services.

Graduate student support was provided by the Laney Graduate School at Emory University and the National Institute of Environmental Health (Award Number 5T32ES12870).

## Ethics statement

The study protocol has been reviewed and approved by institutional review boards (IRBs) or Ethics Committees at Emory University (00089799), Johns Hopkins University (00007403), Sri Ramachandra Institute of Higher Education and Research (IEC-N1/16/JUL/54/49) and the Indian Council of Medical Research—Health Ministry Screening Committee (5/8/4-30/(Env)/Indo-US/2016-NCD-I), Universidad del Valle de Guatemala (146-08-2016) and Guatemalan Ministry of Health National Ethics Committee (11-2016), Asociación Benefica PRISMA (CE2981.17), the London School of Hygiene and Tropical Medicine (11664-5) and the Rwandan National Ethics Committee (No.357/RNEC/2018), and Washington University in St. Louis (201611159). The study has been registered with ClinicalTrials.gov (Identifier NCT02944682).

HAPIN IRB info:

**Table:** 

Institution	Humans subjects protocol number
Emory	00089799
SRU	IEC-N1/16/JUL/54/49
ICMR	5/8/4–30/(Env)/Indo-US/2016-NCD-I
JHU formative	00007464
JHU main	00007403
PRISMA formative	CE 3571.16
PRISMA main	CE3 2981.17
LSHTM	11664-5
RNEC	00001497
UVG	146-08-2016
GMOH	11-2016
Wash U	201611159

## Data availability

Data described in the manuscript, code book, and analytic code will be made available upon request.
