# Supplementary material for: Human Milk Composition Is Associated with Maternal Body Mass Index in a Cross-Sectional, Untargeted Metabolomics Analysis of Human Milk from Guatemalan Mothers
Source: Curr Dev Nutr. 2024 Apr 10;8(5):102144. doi: 10.1016/j.cdnut.2024.102144 (PMC11079463; doi:10.1016/j.cdnut.2024.102144)
Supplement: Multimedia component 1 [file mmc1.docx]

Human Milk Composition is associated with Maternal Body Mass Index in a Cross-Sectional, Untargeted Metabolomics Analysis of Human Milk from Guatemalan Mothers

Kasthuri Sivalogan

**Supplementary Materials**

**Supplementary** **Table S1**. Sociodemographic table for HAPIN, HAPIN-Guatemala and pilot sample participants

|  | HAPIN  N=3,195 | | HAPIN-Guatemala  N=800 | | Pilot Sample  N=75 | |
| --- | --- | --- | --- | --- | --- | --- |
|  | Intervention  N=1,590 | Control  N=1,605 | Intervention  N=400 | Control  N=400 | Intervention  N=38 | Control N=37 |
| Age (years) |  | | | | | |
| <20 | 189 (11.9%) | 209 (13.0%) | 64 (16.0%) | 58 (14.5%) | 9 (23.7%) | 6 (16.2%) |
| 20 - 24 | 616 (38.7%) | 579 (36.0%) | 168 (42.0%) | 156 (39.0%) | 17 (44.7%) | 17 (46.0%) |
| 25-29 | 500 (31.4%) | 517 (32.2%) | 110 (27.5%) | 121 (30.3%) | 9 (23.7%) | 11 (29.7%) |
| 30-35 | 285 (17.9%) | 300 (18.7%) | 14.4 (3.0%) | 14.2 (3.1%) | 3 (7.9%) | 3 (8.1%) |
| Missing | 0 | 0 | 1 (0.3%) | 0 | 0 | 0 |
| Gestational age (weeks), mean (SD) | 15.5 (3.1%) | 15.3 (3.2%) | 14.4 (3.0); 1 | 14.2 (3.1) | 14.9 (2.5) | 14.4 (3.2) |
| Nulliparous^2^, N (%) | | | | | | |
| Yes | 639 (40.2%) | 589 (36.7%) | 119 (29.8%) | 108 (27.0%) | 10 (26.3%) | 8 (21.6%) |
| No | 947 (59.6%) | 1,014 (63.2%) | 281 (70.3%) | 292 (73.0%) | 28 (73.7%) | 29 (78.4%) |
| Missing | 4 (0.3%) | 2 (0.1%) | 1 (0.3%) | 0 | 0 | 0 |
| Highest level of education completed, N (%) | | | | | | |
| No formal education / some primary school | 481 (30.3%) | 558 (34.8%) | 189 (47.3%) | 192 (48.0%) | 13 (34.2%) | 16 (43.2%) |
| Primary school / some secondary school | 558 (35.1%) | 533 (33.2%) | 160 (40.0%) | 152 (38.0%) | 21 (55.3%) | 12 (32.4%) |
| Secondary / Vocational /Some University | 550 (34.6%) | 514 (32.0%) | 51 (12.8%) | 56 (14.0%) | 4 (10.5%) | 9 (24.3%) |
| Missing | 1 (0.1%) | 0 (0.0%) | 1 (0.25%) | 0 | 0 | 0 |
| Height (cm), mean (SD); N missing | 152.1 (6.2); 8 | 152.3 (6.0); 4 | 148.6 (5.0); 1 | 148.2 (5.7) | 149.0 (4.7) | 146.4 (9.4) |
| Body mass index (kg/m^2^) mean (SD); Missing | 23.3 (4.1); 12 | 23.1 (4.0); 7 | 23.9 (3.4); 3 | 23.7 (3.3); 2 | 24.4 (3.8) | 24.1 (4.7) |
| Minimum dietary diversity at six-months postpartum, Category (score) N (%) | | | | | | |
| Low (<4) | 890 (56.0%) | 906 (56.4%) | 279 (69.8%) | 268 (67.0%) | 29 (76.3%) | 29 (78.4%) |
| Medium (4-5) | 496 (31.2%) | 533 (33.2%) | 104 (26.0%) | 115 (28.8%) | 8 (21.1%) | 7 (18.9%) |
| High (>5) | 203 (12.8%) | 165 (10.3%) | 16 (4.0%) | 17 (4.3%) | 1 (2.6%) | 1 (2.7%) |
| Missing | 1 (0.1%) | 1 (0.1%) | 1 (0.3%) | 0 | 0 | 0 |
| Household food insecurity, Category (score), N (%) | | | | | | |
| Food secure | 930 (58.5%) | 863 (53.8%) | 215 (53.4%) | 324 (81.0%) | 19 (50.0%) | 21 (58.3%) |
| Mild (1, 2, 3) | 416 (26.2%) | 448 (27.9%) | 126 (31.5%) | 129 (32.3%) | 14 (36.8%) | 10 (27.8%) |
| Moderate (4, 5, 6) / Severe (7, 8) | 220 (13.8%) | 272 (16.9%) | 43 (10.8%) | 52 (13.0%) | 5 (13.2%) | 5 (13.9%) |
| Missing | 24 (1.5) | 22 (1.4) | 6 (1.5%) | 4 (1.0%) | 0 | 1 |

**Supplementary Table S2**. Mummichog output of pathway analysis of maternal BMI

| Pathways | Overlapping features^α^ | Metabolites in pathway^β^ | P-value | Technical column |
| --- | --- | --- | --- | --- |
| Sialic acid metabolism | 19 | 33 | 0.0009 | HILIC |
| Biopterin metabolism | 12 | 20 | 0.0012 | HILIC |
| Hexose phosphorylation | 10 | 19 | 0.0030 | HILIC |
| Tyrosine metabolism | 40 | 98 | 0.0046 | HILIC |
| N-Glycan degradation | 5 | 8 | 0.0054 | HILIC |
| Ascorbate (Vitamin C) and aldarate metabolism | 10 | 21 | 0.0063 | HILIC |
| Galactose metabolism | 17 | 41 | 0.0106 | HILIC |
| Aminosugars metabolism | 16 | 39 | 0.0129 | HILIC |
| Glycosphingolipid biosynthesis (globoseries) | 5 | 10 | 0.0184 | HILIC |
| Keratan sulfate degradation | 5 | 10 | 0.0184 | HILIC |
| Purine metabolism | 24 | 63 | 0.0233 | HILIC |
| Linoleate metabolism | 9 | 22 | 0.0274 | HILIC |
| Vitamin H (Biotin) metabolism | 3 | 5 | 0.0286 | HILIC |
| Lysine metabolism | 12 | 31 | 0.0335 | HILIC |
| Starch and sucrose metabolism | 8 | 20 | 0.0381 | HILIC |
| Glycosphingolipid metabolism | 15 | 40 | 0.0394 | HILIC |
| Fatty acid metabolism | 9 | 18 | 0.0032 | C18 |
| Glycerophospholipid metabolism | 16 | 45 | 0.0055 | C18 |
| De novo fatty acid biosynthesis | 10 | 26 | 0.0071 | C18 |
| Hexose phosphorylation | 8 | 20 | 0.0086 | C18 |
| Carbon fixation | 5 | 10 | 0.0087 | C18 |
| Vitamin B6 (Pyridoxine) metabolism | 4 | 7 | 0.0095 | C18 |
| Linoleate metabolism | 8 | 22 | 0.0146 | C18 |
| Lysine metabolism | 10 | 30 | 0.0176 | C18 |
| Vitamin B1 (Thiamin) metabolism | 5 | 12 | 0.0185 | C18 |
| Fatty acid activation | 8 | 23 | 0.0192 | C18 |
| Phosphatidylinositol phosphate metabolism | 10 | 31 | 0.0224 | C18 |
| Limonene and pinene degradation | 3 | 6 | 0.0342 | C18 |

^α^Number of metabolites with m/z matched to the enriched metabolites in the specific pathway

^β^Total number of metabolites enriched in the specific pathway

**Supplemental Figure 1**. Significant pathways and number of significant metabolites within pathways associated with maternal BMI using both positive- and negative-ion mode data. Pathways with p≤0.05 and number of significant metabolites ≥1 are included. Point size indicates the number of significant metabolites identified within each pathway. All pathways have the same local false discover rate (lfdr) [38].

**
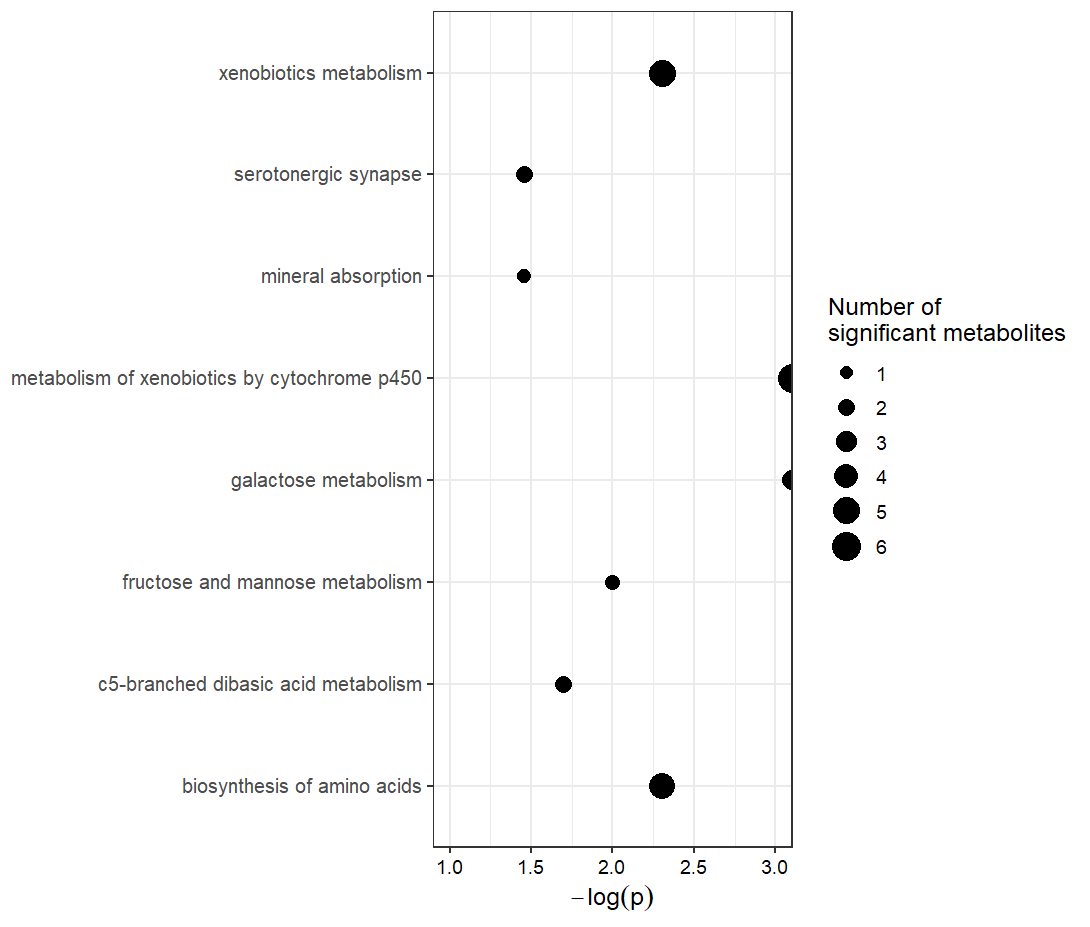
**
